# Supplementary material for: ‘You don’t have to sleep with a man to get how to survive’: Girl’s perceptions of an intervention study aimed at improving sexual and reproductive health and schooling outcomes
Source: PLOS Glob Public Health. 2022 Oct 13;2(10):e0000987. doi: 10.1371/journal.pgph.0000987 (PMC10021241; doi:10.1371/journal.pgph.0000987)
Supplement: S1 Checklist — (DOCX) [file pgph.0000987.s001.docx]

Inclusivity in global research

PLOS’ policy on inclusivity in global research aims to improve transparency in the reporting of research performed outside of researchers’ own country or community and ensures that PLOS publications reporting global research adhere to high standards for research ethics and authorship. Authors of relevant research articles may be asked to complete the questionnaire below, which outlines ethical, cultural, and scientific considerations specific to inclusivity in global research. This questionnaire may be requested when researchers have travelled to a different country to conduct research, if research uses samples collected in another country, research with Indigenous populations or their lands, or if research is on cultural artefacts. Researchers travelling to another country solely to use laboratory equipment will not normally be required to complete the questionnaire. However, the questionnaire can be requested at the journal’s discretion for any submission – if you have been requested to complete this questionnaire by the PLOS journal you submitted to, please do so.

Please complete the questionnaire below and include this as a Supporting Information file with your manuscript. Note that if your paper is accepted for publication, this checklist will be published with your article in the supporting information files. Please ensure that you reference the checklist in the main body of your manuscript. We suggest adding a subsection ‘Inclusivity in global research’ to your Methods section and adding the following sentence: “Additional information regarding the ethical, cultural, and scientific considerations specific to inclusivity in global research is included in the Supporting Information (SX Checklist)”

The questions have been designed to be applicable to a wide range of study types, and there are subsections for both human subjects research and non-human subjects research. If any of the questions are not relevant to your research please mark them as “N/A” as appropriate.

**Ethical considerations, permits and authorship**

*This section is applicable to all research types.*

Provide details as to who granted permissions and/or consent for the study to take place in the Methods section of your manuscript. This should include the names of **all** ethics boards, governmental organizations, community leaders or other bodies that provided approval for the study. If individuals provided approval refer to these people by their role or title but do not list their name(s).
1. Kenya Medical Research Institute (KEMRI) Scientific Ethics Review Unit (SERU) 3215

Reported on page number:10

2. Liverpool School of Tropical Medicine (LSTM) Research Ethics Committee (REC)15-005

3. Centres for Disease Control (CDC) Institutional Review Board (IRB) 2016-136

4. Pharmacy and Poisons Board- Kenya (KPPB) ECCT/16/07/06

5. Ministry of Education, Science and Technology Ref. No. MOE.HQS/E/6/36

If there were any deviations from the study protocol after approval was obtained please provide details of these changes in the Methods section of your manuscript.

Reported on page number:

A review of filing documentation on 3 November 2017 uncovered an error in the implementation of the allocated intervention in one of the 84 schools. Two schools in two different sub-counties have the same name and had been allocated to into two different arms – one into cash only, and one into cash and cups, at randomization. However, the same name caused an error with both schools receiving interventions for both cash and cups. The school that received cups as an additional intervention in error comprised 39 participants. All parental consents and girls’ assents completed were also for agreement to receive both cash and cup interventions, and training, safety guidelines and support were provided as per protocol for this.

No adverse events have been reported following provision of interventions among the 39 participants.

There was a delay in the release of HIV results to study participants within a month as stated in the study protocol. Of 4125 participating girls in 96 schools tested, there was a miscalculation of return date of results for participants in one school meaning results for 57 girls were 2 weeks late.

HIV tests were conducted on 3004 participants at baseline in the year 2017 using the following algorithm: first a Determine™ rapid test would be used, if Determine™ was negative then a negative result would be reported. If Determine™ tested positive, then a confirmatory test would be performed on the sample using the Unigold™ kit. If the Unigold™ test yielded a positive result, then this sample would be identified as HIV positive. If the Unigold™ test yielded a negative result then the sample would be tested a third time with an Elisa Biorad third generation™ tie-breaking test. The Biorad™ result would be the final conclusive HIV result for the sample. All positive results in 2017 were in accordance to this testing algorithm. These results were then reported to the participants’ respective health facilities and the participants received their HIV results from the facility point persons at the designated Comprehensive Care Clinics. In the 2018 follow up round of the study, all 2017 participants still enrolled in school were tested again according to the study protocol. Their blood samples were again tested in accordance to the testing algorithm described above. Six participants, who had been reported as HIV positive in 2017, were found to be HIV negative in 2018. The negative results of 2018 were then provided to the health facility for subsequent relay to the study participants. When the study realized that the participants had received conflicting results, all samples for these participants from both 2017 and 2018 were retested using PCR and yielded negative results. Ministry of Health (MOH) guidelines require that health facilities repeat HIV tests and confirm positivity before initiation of HIV care and treatment at the facility and thus all these participants had not been initated into care based on our results. They were retested at the MOH facility and their results were reported negative. These results were relayed to them and counselling was done to the participants. The HIVcouselling and testing counsellors also explained to them why the anomaly may have occurred.

Samples were collected on DBS 903 filter paper to test for DNA PCR in the event that the test results done via rapid test kits were discordant according to the study’s SOP. In three cases, samples were delivered to the lab by research staff and subsequently misplaced; they could not be found to be tested. In one case a sample accidentally fell into 10% hypochlorite solution during sample preparation. Recollection for the mentioned samples was done and was tested and dispatched to the field. Thus, for these four cases the final HIV results took longer to be delivered to the participants. To the best of our knowledge no adverse events have been reported following this delay. The participants received communication about this delay. Sample recollection was done with the permission of the participants.

Did this study involve local collaborators that are residents of the country where the research was conducted or members of the community studied? If you do not have any authors from said communities, please provide an explanation for this below.

Everyone listed as an author should meet PLOS’ criteria for authorship and all individuals who meet these criteria should be included in the author byline, rather than the acknowledgements. Authorship criteria is based on the International Committee of Medical Journal Editors (ICMJE) Uniform Requirements for Manuscripts Submitted to Biomedical Journals - for further information please see here: <https://journals.plos.org/plosone/s/authorship>.

Yes the study did involve local collaborators. Three of the authors are local to the study area:

Elizabeth Nyothach MA (Enyothach@kemri.go.ke);

David Obor, MSc^3^ ([DObor@kemri.go.ke](mailto:DObor@kemri.go.ke));

Eunice Fwaya, BPharm^3^ ([eunicefwaya@gmail.com](mailto:eunicefwaya@gmail.com));

Our longstanding collaboration between LSTM and KEMRI means that overseas researchers are immersed in the local culture, whilst key members of our research team and the majority of our field staff comprise of local Kenyans.

**Human subjects research (e.g. health research, medical research, cross-cultural psychology)**

Did you obtain written informed consent from a representative of the local community or region before the research took place? How did you establish who speaks for the community? Details of written informed consent obtained from study participants should be reported separately in the Methods section of your manuscript.

Prior to the study taking place representatives from our research team met with education officials and chiefs at the county and subcounty levels and gave them an overview of the study, provided information, answered questions. The education officials were very helpful in mobilizing head teachers and local community members. Chiefs were also very useful in mobilizing community members during their meetings. Written informed consent was not requested at this point. A public ceremony was held for the randomisation process, with head teachers or representatives from all schools participating in the event. Head teachers provided written consent. Informed consent was sought from parents/guardians of the school girls then informed assent was requested from girls whose parents/guardians had consented. The staff who were recruited from the local community were mainly responsible for facilitating these meetings.

How did members of the local community provide input on the aims of the research investigation, its methodology, and its anticipated outcome(s)?

The study held meetings with different stakeholders including the parents/guardians, local teachers, local health and ministry official, education officials, chiefs and community health volunteers. During these meetings the community members were given a chance to ask questions and give their opinions about the study.

When engaging with the local community, how did you ensure that the informed consent documents and other materials could be understood by local stakeholders?

The informed consent documents were translated into mother tongue (Dholuo) and Swahili (national language). Field staff who understand the local language were recruited from the local area. This ensured that they could be able to communicate with the people in the community. The community members would be asked the language that they are comfortable with and this would be used in communication.

Will the findings of the research be made available in an understandable format to stakeholders in the community where the study was conducted (e.g. via a presentation, summary report, copies of publications, etc.)? Please provide details of how this will be achieved.

When the study is completed the study will do summary reports in national and local languages and disseminate the finding through barazas (meeting at the local community) that are convened by chiefs. Fliers will also be distributed to the education offices at the county and subcounty offices.

**Non-human subjects research using specimens/ animals collected as part of the study, or those housed in archival collections. Examples include archaeology, paleontology, botany and zoology.**

Did the permission you obtained from a local authority to perform the study include an agreement on access to outputs and benefit sharing? This may include procedures to enable fair distribution of the benefits and resources arising from the research performed. Please include any details of Prior Informed Consent and Benefit Sharing Agreements obtained. These may be required by field-specific regulations, for example the Convention on Biological Diversity (CBD) and the associated Nagoya Protocol.

N/A

If the material used in your study was imported, please A) provide the year it was imported and B) indicate whether permits were obtained to import/export the materials used, C) provide details of any permits obtained. If this information is not available, please indicate this.

Menstrual Cups (Mooncup) were imported from the UK from 2016 to 2022. Certificate of analysis was obtained during shipment of the Mooncups. Permit for shipment was also obtained from Jambo logistics.

If you used archival specimens, please state how the material used in your study was acquired by the institute it is held in and provide details of any permits obtained for the original excavations/ sample collection. If this information is not available, please indicate this.

N/A

How was the potential cultural significance of the materials collected in your study to local communities considered in your research design? Were Indigenous peoples and/or local researchers and institutions involved with archaeological excavations / collection of specimens? If so, please provide a description of their involvement.

N/A

If your manuscript includes photographs of human remains please indicate whether authors obtained permission from descendants or affiliated cultural communities to do so.

N/A
