# Supplementary material for: ‘You don’t have to sleep with a man to get how to survive’: Girl’s perceptions of an intervention study aimed at improving sexual and reproductive health and schooling outcomes
Source: PLOS Glob Public Health. 2022 Oct 13;2(10):e0000987. doi: 10.1371/journal.pgph.0000987 (PMC10021241; doi:10.1371/journal.pgph.0000987)
Supplement: S1 Fig — (DOCX) [file pgph.0000987.s002.docx]

Girls enrolled into the cash intervention

n = 990

Girls enrolled into the cup intervention

n = 990

24 schools randomized to cup intervention

24 schools randomized to a control arm

24 schools randomized to cash intervention

**Cup intervention**

2 schools chosen randomly

FGD = 1 from each

**Cash intervention**

2 schools chosen randomly

FGD = 1 from each

**Control intervention**

2 schools chosen

randomly.

FGD = 1 from each

Figure 1. Flowchart of randomisation and qualitative study design.

**Cup intervention**

2 schools chosen

randomly.

FGD = 1 from each

**Cup intervention**

2 schools chosen

randomly.

FGD = 1 from each

**Intervention**

**Control intervention**

2 schools chosen

randomly.

FGD = 1 from each

**Cash intervention**

2 schools chosen

randomly.

FGD = 1 from each

**Combined intervention**

2 schools chosen

randomly.

FGD = 1 from each

**Cash intervention**

2 schools chosen

randomly.

FGD = 1 from each

**Combined intervention** 2 schools chosen

randomly.

FGD = 1 from each

**Endline FGD**

**Control intervention**

2 schools chosen randomly

FGD = 1 from each

**Combined intervention**

2 schools chosen randomly

FGD = 1 from each

**Midline FGD**

**Randomization**

**Enrolment**

**Baseline FGD**

Girls enrolled into

the combined intervention

n = 990

Girls enrolled into the control arm

n = 990

24 schools randomized to combined cash and cup intervention

96 schools enrolled
