# Supplementary material for: ‘You don’t have to sleep with a man to get how to survive’: Girl’s perceptions of an intervention study aimed at improving sexual and reproductive health and schooling outcomes
Source: PLOS Glob Public Health. 2022 Oct 13;2(10):e0000987. doi: 10.1371/journal.pgph.0000987 (PMC10021241; doi:10.1371/journal.pgph.0000987)
Supplement: S1 Table — (DOC) [file pgph.0000987.s003.doc]

**Supplementary Information 1.1**

**BASELINE FOCUS GROUP DISCUSSION FOR SECONDARY SCHOOLGIRLS**

| MODERATOR: Document required information as appropriate for each FGD using the formats provided below. Date: _____/_______/________  Initials: Moderator: ______ Note Taker______ Recorder Number: ____ Folder/File Name (location on recorder): _______________________  Interview location (Venue): _________________________________________________________  FGD Group: _____________________________________________________________________ FGD Number: _____  Time Start: _______________ Time stop: ________________ No. Participants at start of FGD: ________ No. Participants at the end of FGD: _________  Demographic information for every FGD participant *[to be completed on a one-to-one basis, immediately after consent is obtained]*   | Participant number or Fake name | Age in completed years | Ethnic group | | --- | --- | --- | | 1 |  |  | | 2 |  |  | | 3 |  |  | | 4 |  |  | | 5 |  |  | | 6 |  |  | | 7 |  |  | | 8 |  |  | | 9 |  |  | | 10 |  |  | | 11 |  |  | | 12 |  |  |   COMMENTS – reasons for withdrawal, refusal, ambience of FG, level of interest, disagreements, etc: ___________________________________________ _______________________________________________________________________________________________________________________________  _______________________________________________________________________________________________________________________________  Introduction *[To be read to the participants at the start of the focus group]*  Thank you for your willingness to take part in this informal group discussion. My name is [Name]. I am from KEMRI/CDC. I am here because we are doing research on how to keep girls in secondary school, and we seek your help in understanding the important issues that affect girls both in and out of school. We’re interested in hearing from you which factors result in girls going to school or dropping out of school. Often people from outside think they know what you think regarding these issues when they really don’t. To us, you are the real experts, and there’s a lot we can learn from you. So today I’d like to hear your views about what you or other people in this community and school think. I also want to remind you that everything we talk about today is confidential. No one will hear this tape except for the people working on the research. Whenever we write a report, we will use numbers or fake names so no one can identify you. If there are any questions you’d rather not answer, just let me know - that’s fine. There are no “right” or “wrong” answers to our questions, only what you think. Your answers can be based on your experiences, observations, or feelings. Everyone’s views are equally important. It’s fine to disagree with other girls but if you do, it’s important to disagree in a respectful and polite manner. If you disagree with something anyone says, you can say ‘I disagree’ and then wait for them to finish before you speak. I ask that you all talk in turns, because if you all speak at once, I will not have a clear recording and the study staff will have a hard time understanding the important issues we will talk about today. I will now introduce my colleagues and see if you have any questions for me before we begin.  *[Explain the role of note-takers and tape-recorder; Give a few minutes for answering any questions regarding the informal FGD]*  NOTE GIRLS QUESTIONS HERE: _____________________________________________________________________________________________________________  _______________________________________________________________________________________________________________________________________  _______________________________________________________________________________________________________________________________________ | | |
| --- | --- | --- | --- | --- | --- | --- | --- | --- | --- | --- | --- | --- | --- | --- | --- | --- | --- | --- | --- | --- | --- | --- | --- | --- | --- | --- | --- | --- | --- | --- | --- | --- | --- | --- | --- | --- | --- | --- | --- | --- | --- |
| MODERATOR: *[This is focus group is a conversation between the girls for them to help us better understand issues. This will inform a large study in secondary schools. It is not meant to be only about menstruation, but about all the things that affect schoolgirls. We want to know the main factors, from the girls’ perspective, that stop girls from enjoying school, engaging and completing secondary school.]* | | |
| Themes | Questions | Prompts |
| Warm up and overview | Tell me about school…  Do you enjoy school?  Why do you attend school?  What about girls your age? What do they think of staying at school? | Why? Why not?  Reason: lack alternatives / qualifications / future employment / made to attend  Is it your choice to be at school? Does your family support you in your choice?  What work do girls do instead of going to school and finishing their education? |
| Absenteeism | What causes absenteeism?  Do girls fall behind with schoolwork if they miss school?  What happens when girls miss school often? | Why do they fall behind? What happens if they fall behind in class –  Is there a way to catch up once girls fall behind?  Do they attend school less and less? What do they do? |
| Experience and challenges around commitments at home | Does having to do chores stop you or other girls from going to school?  Do you or other girls find it difficult to do assignments / homework because they have to do chores?  Can girls who are orphans complete secondary school? | Does this happen often or occasionally? Is it most girls or just some?  Which chores stop girls from doing assignments? What happens?  Why not? Do you know orphaned girls, are there many here? who cares for them, pays their fees, what are their problems? |
| Experience and challenges around menstruation | What do you /other secondary school girls use to manage menstrual periods?  Where can you get them (pads / tampons / cups) from? Do they cost a lot?  Do girls struggle managing their periods at school?  Does menstruation play an important role in taking days off secondary school (being absent)?  Does menstruation play an important role in not concentrating or taking part in school work/activities?  Does menstruation play an important role in girls dropping out of school? | List what they use and problems/challenges.  If pads are given in school, how many/month? Is it enough? Are they good quality pads?  If pads not from school, how do you obtain them? Do you buy them with your own money? Does someone buy them for you?  Do girls get pads from sexual partners? Do girls give sexual favours in return for pads or money to buy pads?  What problems do girls have? – leaking; other (cramps, pads, privacy, soap, water, disposal, concentration, smell, teasing)  Do secondary school girls get more help than primary girls for managing periods? If so, what is this? Why does this happen?  To what extent does menstruation affect not going to school? Does it happen to most girls / a few girls? Do girls not attend every month / occasionally?  What is the main reason for not being able to concentrate (is it physical – i.e. cramps) or because of poor management (i.e leaking, smell)?  To what extent does menstruation affect dropping out of school? Does it happen to most girls / a few girls? |
| Experience, challenges and consequences around travel and time | If girls have to travel a long distance to and from school – what effect does this have? Do girls arrive to school late, or have less time for chores?  Do girls ask boda-boda drivers for lifts?  What can make some girls oversleep?  Do girls take time off school for funerals (of relatives)? | How do girls get to school? Are there people who help you get to school? If yes who?  Do they give girls lifts to/from school? How often? Do girls like this? What do girls give in return? Do boda-boda operators give gifts? (what are they?). If girls do not accept from boda-boda, why not?  PROBE reasons for oversleeping, (chores / homework / relationships) and how often this occurs.  How often do girls have to help family with funerals?  What about afterwards (disco-funerals…..late nights / sex) |
|  |  |  |
| Experience and challenges around money | Who **generally** pays **girls** school fees? What are other school costs that are expensive and make it hard for girls to attend school?  How do **secondary school girls** usually get money?  What sort of things do **girls** spend the money on?  **Are many girls** able to save money?  **Do girls often have to give some of their money away?**  **We have heard that** some girls may receive money from boyfriends / males after having sex with them. Do you think this happens to girls of your age?  **Instead of receiving money** – might girls receive gifts from sexual partners? | Apart from parent/guardian do others help? Can girls or parents get the money from other people – who? Do girls drop out if family cannot afford?  What are the circumstances? **What sort of things do girls do to earn money?**  **Is this for necessities or is this for non essentials**?  Is this to help the family or for their own things?  What do **girls** NEED but can’t afford?  What sort of things do **girls** WANT but can’t afford?  Is this difficult? In what ways?  If so, describe circumstances, (who / why / how much?) Is this for any money earnt or just for doing certain things (what?)  Is this **generally** expected? Is this ok?  **What are your thoughts about this?**  Who are the boyfriends? Is it OK to have men as partners; or multiple partners? What sort of things might the money be used for? (Prompt for essentials or non-essentials)  Is it many girls or just a few (which type of girls – poor background / orphans/struggling to stay at school/those who want to get married…..?)  How do you feel about this? (normal / acceptable / only way….?)  Can you tell us about this…..(What kind of gifts? Is this a good thing for girls? Why?  Do girls get different gifts or things from different men? Are there special names for different men/boyfriends according to the gifts they give?  **Does this happen often?** |
| Experience and challenges around sex | Do secondary school girls **usually** have sexual relationships?  Why **might girls of your age** first have sexual activity?  **Is pregnancy common in girls** attending secondary school?  What happens when girls get pregnant?  What are consequences of getting pregnant?  Is pregnancy main reason girls get married? | Prompt if need: (1) they want to anyway (2) they want to please their boyfriend, (3) they want to get money or gifts, (4) because everyone is doing it 5) they were tricked / forced (*if they allude to this, prompt further – is this common? Who might trick or force them? Do you know how this might happen?*)  **Can you tell us about this?** (Does this happen a lot? Can you say anything about the availability and choice of protection for girls of your age?)  How do they find out they are pregnant; are there things done by the school administration to check if the girls are pregnant? Who can girls go to for help?  How does the school or parent respond if a girl becomes pregnant? Does the girl have to stop going to school?  What are reasons girls leave school to marry and how common is this? |
| Dropout | Do you and your friends ever think about or talk about dropping out of school?  Is there pressure on girls to leave school?  What about your peers who do not attend school anymore – why do you think they dropped-out?  Do you think girls who drop out of school are similar or different to girls like yourselves who stay at school? | If yes, why do they think about it?  Who is exerting the pressure? What happens?  What are the main reasons they drop- out? Out of the things mentioned, what is MAIN reason? The second most important; the third ….  If disagreement between girls, what other things do some girls say are very important.  If yes is this frequent – more in girls than boys? Why? Do you think they wanted to stay at school if not: why not? If yes: why can’t they? Did they have a choice? |
| Preventing dropout | What can be given or done to help girls stay in school to complete their education (and sit in final exams)?  Would menstrual support stop girls from dropping out?  Would financial support stop girls from dropping out? | Let girls give their views. List things girls say.  What menstrual support would keep girls in school – how would it help - would this be enough alone or what other things are needed to keep girls in school?  What would this be used for? |
