# Supplementary material for: ‘You don’t have to sleep with a man to get how to survive’: Girl’s perceptions of an intervention study aimed at improving sexual and reproductive health and schooling outcomes
Source: PLOS Glob Public Health. 2022 Oct 13;2(10):e0000987. doi: 10.1371/journal.pgph.0000987 (PMC10021241; doi:10.1371/journal.pgph.0000987)
Supplement: S2 Table — (DOCX) [file pgph.0000987.s004.docx]

| **MODERATOR:** Document required information as appropriate for each FGD using the formats provided below. Date: _____/_______/________  Initials: Moderator: ______ Note Taker______ Recorder Number: ____ Folder/File Name (location on recorder): _______________________  Interview location (Venue): _________________________________________________________  FGD Group: _____________________________________________________________________ FGD Number: _____  Time Start: _______________ Time stop: ________________ No. Participants at start of FGD: ________ No. Participants at the end of FGD: _________  **Demographic information for every FGD participant *[to be completed on a one-to-one basis, immediately after consent is obtained]***   \| **Participant number or**  **Fake name** \| **Age in completed years** \| **Ethnic group** \| \| --- \| --- \| --- \| \| 1 \|  \|  \| \| 2 \|  \|  \| \| 3 \|  \|  \| \| 4 \|  \|  \| \| 5 \|  \|  \| \| 6 \|  \|  \| \| 7 \|  \|  \| \| 8 \|  \|  \| \| 9 \|  \|  \| \| 10 \|  \|  \| \| 11 \|  \|  \| \| 12 \|  \|  \|   **COMMENTS – reasons for withdrawal, refusal, ambience of FG, level of interest, disagreements, etc** |
| --- | --- | --- | --- | --- | --- | --- | --- | --- | --- | --- | --- | --- | --- | --- | --- | --- | --- | --- | --- | --- | --- | --- | --- | --- | --- | --- | --- | --- | --- | --- | --- | --- | --- | --- | --- | --- | --- | --- | --- |
|  |
| **Introduction**  Thank you so much for your willingness to take part in this group discussion. My name is **[Name]**. I am from the KEMRI. We are doing a research study  to see if we can help prevent girls from dropping out of school early  and keep them healthy as well.  We’re interested in hearing from you about what it is like to be a schoolgirl, at **[Name of community/school]** and whether being part of the study has made any difference to you and your peers.  Often people from outside think they know what you think regarding these issues when they really don’t. To us, you are the real experts, and there’s a lot we can learn from you.  So today we would like to hear your views . This is very informal; you can talk about anything you think is important for us to know. I also want to remind you that everything we talk about today is confidential.  No one will hear this tape except for people working on the project. Whenever we write a report, we will use numbers or fake names so no one can identify you.  If there are any questions you’d rather not answer, just let me know - that’s fine.  Your frank responses and discussion will be most helpful to us as we try to really understand these issues. Remember, your answers to our questions will not be considered “right” or “wrong”, because we want to know about what people think. They are merely information you will provide based on your experiences, observations, or feelings. Everyone’s views are equally important. It’s fine to disagree with other people’s views, but if you do, it’s important to disagree in a respectful and polite manner. It’s important for you to talk in turns to speak, because if you all speak at once, we will not have a clear recording. If you disagree with something anyone says, you can say ‘I disagree’ and then wait for them to finish before you speak.  **Explain the role of note-takers and tape-recorder**  **Give a few minutes for answering any questions regarding the FGD**  Please note the questions here:  ______________________________________________________________________________________________________________________________  ______________________________________________________________________________________________________________________________  ______________________________________________________________________________________________________________________________  ______________________________________________________________________________________________________________________________  ______________________________________________________________________________________________________________________________ |

**GIRLS ENDLINE FGD – CONTROL GROUP**

| Remember this needs to be a discussion amongst the girls. Your role is to introduce the topics and steer them if they go off topic, otherwise let it flow. Use your judgment when you need to prompt – bear in mind what is really important for us to know. Don’t forget to do a really good ice breaker to get them chatting before you start. |
| --- |

Information / Instructions Key Topics / Questions Probes

| So this is just to make sure they are really aware and not talking about a different intervention. Make sure they clearly understand that it is our study being discussed and not another intervention that is happening in school or community. These girls will have received puberty hygiene education, hand soap, bloods taken for HIV and survey participation. Also be aware that they might not have noticed much or have much of an opinion so don’t force them to come up with answers if they can’t really think of anything. Get key responses, find out level of agreement generally but don’t go round the room so that everyone has to say something. Just draw out if shy and look like they want to contribute but louder girls are overpowering them. If girls mention anything that we would have asked about later in this FGD, you can dig in here and gather all the information then miss out later, or ask later – but you don’t need to ask twice. | Lets talk about the KEMRI study first. What did you think about being in the study?  Was there anything you didn’t like about the study? If yes; can you tell us about this and why you didn’t like it.  Was there anything you did like about the study? If yes: Can you tell us about this and why you liked this.  Since the study began have you felt differently about coming to school? Do you enjoy it less or more?  Do you feel that girls in your form have changed at all because they have been part of the study?  Do you feel that other people’s attitudes towards girls in the study have changed because they were In the study? | Probe to find out level of agreement. Were these key issues for many or just the odd person.  Probe to find out level of agreement. Were these key issues for many or just the odd person.  Explore the reasons why  Probe to find out what changes have occurred and why they think it resulted from being in this study.  Probe to find out who/what sort of people. Check family / friends/boyfriends/older men / boda boda…anyone else?  For each group find out how their attitudes have changed towards the girls and why this was because the girls were in the study. |
| --- | --- | --- |
| This section is on schooling and the study. One of the key outcomes for the study is school drop-out so here we will focus on whether this study might have helped prevent any drop-out.  For any question where they don’t have much to say then move on rather than try and force them to give a response. Concentrate on questions that they can give good information on. | Tell us about school drop out…have many girls who enrolled into secondary school in your year have given up schooling? Why did they leave school?  Thinking back, have you noticed any differences in the number of girls who drop out in the early forms – 1 & 2, compared to later - forms 3 or 4?  Do you think girls who drop out of school are very different to girls like you who stay until form 4?  Do you think being in this study has made any difference to girls of your age, staying in school….or leaving school? | If yes – find out how many / what were key reasons for drop-out if known. (probe pregnancy/fees/peer pressure/absence etc) What can be done to prevent this drop-out?  Probe to get an explanation if they say yes – how and why. (If they haven’t noticed anything then move on) Are there differences in the reasons for drop-out over time?  Probe to get an explanation of their answers as to why (if any difference is identified)  Probe to get an explanation of their answers as to why (if any difference is identified) |
| Now move to find out about school fees, attendance and performance, and whether being in the study might have some effect on these. We already know quite a lot about these so this is just to allow comparison across the different study arms – and also any changes in this group due to being in the study  For all questions that you follow up, find out if this is the opinion of most of the group or just one or two opinions. Consider asking ‘does anyone agree or disagree with this’  Also find out if many or just a few girls are affected. | We have seen that girls are sent home for fees, how long is it usually before they are allowed back into school? Are they able to continue as normal or does this affect their schooling in any way?  Do many girls have to repeat the form again?  Is there anything that makes it difficult for girls like yourselves to work in class and do well in your studies?  Thinking back to when you first started in the study, has anything changed since then because of being in the study which makes it easier or more difficult for you to work in class and do well in your studies?  Moving on to school absence - Do many girls in your year miss school often?  Thinking back to when you started being in the study, do you think there have been any changes in girls’ ability to attend school as a result of being in the study?  Tell us about your relationships with the teachers at your school – are they supportive of schoolgirls like yourselves?  So, again thinking back to when the study started, have teachers attitudes or behaviour towards you changed at all or has it remained pretty much the same? | Find out how long they tend to be away from school, whether they return, how they catch up and whether this has any effect on them in the short or long term.  If this is common find out why they have to and how it affects these girls. Find out if teachers support these girls or whether they make things more difficult.  Find out what, if anything does and why.  If they identify anything then find out what this is and why it has changed as a result of the study  If so, find out whether many or just a few girls miss school and what the key reasons are. Do not push them to talk about menstruation – let them give the different reasons  Find out what these changes were, why they happened i.e how they were due to the study, when did they happen (was this a gradual/sudden change, over the last few months or at the start of the study etc)  If not, explore why girls think this  If changes, then explore how and why |
| This section of the study is to find out if being in the study has made any difference to how girls feel about themselves (e.g. self-esteem and confidence) | Do girls in your year feel shy or confident when they are in school? Why is this?  What about being around boys in your year….Do you think girls in your year are confident or shy in dealing with these boys? Why is this?  Do you think there have been any changes in girls confidence in the 2 years or so since being in the study? Do you think these changes are a result of being in the study? | Find out if girls feel able to say ‘no’ and stand up to boys, whether boys can pressure or ‘bully’ them, or whether it is the other way around and girls can make boys do what they want. If so, how do they manage this?  If they suggest any changes are because of the study then find out what these are and why they have happened |
| We also want to know if being in the study has made any difference in girls relationships with boys or men, particularly in relation to having sex for money or gifts.  Some of this information may have already come out from the questions above, so just ask and probe once. | Do girls in your year have relationships with boys or men? Tell us what sort of relationships they are….  *If they describe sexual relationships*, ask: we have heard that some girls have sex for gifts or money, do you think this happens to other girls in your forms (in your school, or other schools you know of) ?  Which schoolgirls – all or just some? What makes some girls need to do this but not others? What are the differences between these girls?  Do you think that relationships girls in the study have with boys / men has changed at all because of the study? | Probe to find out if they are just friendships, sexual or a mix. Are they mostly with boys of their own age, or with older men. Do girls have multiple relationships, or serial (i.e they move on from one straight to the next). Do girls really want these relationships or are they pressured (if so, why and how)  Probe to find out what they receive, gifts or money –if cash what do they use the money for**** (NB this is important)  Probe to find out if girls think this is a good situation – are they in control of the boys and it is their choice, or do they do this out of real need / desperation. Is it common to have many different boyfriends or do they stick to just one? Do they want this situation to change? If yes: how could it be changed?  If yes, probe to find out in what way they have changed, how this relates to being in the study (if at all), and when these changes occurred, and how quickly or gradual they were.  If not /not really just move on |
| Questions on cash which will be useful for comparing with girls in the other arms of the study.  Only ask these questions if they haven’t already been covered. | Are girls like yourselves able to get and save money?  What sort of things do girls in your year need money for?  If you received 500/ a month, what would you use it for? | If yes, find out how they get money what they use it for, or if they save - what is this saved for?  Find out if they think this would be enough for their needs, and if not, why not |
| Questions on menstruation which will be useful for comparing with girls in other arms of the study. Also bear in mind that girls in the control group will have received puberty & hygiene training and some soap – and will be given a cup at the end, so there may have been some changes or difference expectations because of the study already.  As before only ask these questions if these haven’t already been covered | Are there any other programmes in your community that give pads or money to families to help girls  Does your school give sanitary pads to girls?  What are girls in your year using to manage your periods?  Has this changed in the years since starting this study?  Do girls like yourselves find menstruation easy to manage? Why / why not? What would help?  Have any of you heard of the menstrual cup? | If yes, find out more – DREAMS or others – in your form are there many girls supported with this? how many? How does it help them? Do you think it makes any difference to their schooling? What about their lives generally?  If yes, find out more - Is this to all girls, or just if they need one in an emergency? If to all girls – how many do they give out? How often? Are they enough? What are the pads like? What is good about this? What is not good about this?  Find out what they think of these materials. If pads, how do they afford them?  Find out what any changes are and why they have changed.  If yes, find out what they know about it and what they think. |

**GIRLS CUP GROUP ENDLINE FGD**

Information / Instructions Key Topics / Questions Probes

| So this is just to make sure they are really aware and not talking about a different intervention. Make sure they clearly understand that it is our study being discussed and not another intervention that is happening in school or community. These girls will have received puberty hygiene education, hand soap, bloods taken for HIV and survey participation. most will have received a menstrual cup so it is likely that they will discuss this as part of liking/ not liking the study. If they bring this up themselves then dig deep here and find out all the cup information now rather than later. If they do not mention it, then wait until the section specifically on the cup to ask them  Also be aware that they might not have noticed much or have much of an opinion so don’t force them to come up with answers if they can’t really think of anything.  Get key responses, find out level of agreement generally but don’t go round the room so that everyone has to say something. Just draw out if shy and look like they want to contribute but louder girls are overpowering them.  If girls mention anything that we would have asked about later in this FGD, you can dig in here and gather all the information then miss out later, or ask later – but you don’t need to ask twice. | Lets talk about the study first. What did you think about being in the study?  Was there anything you didn’t like about the study? If yes; can you tell us about this and why you didn’t like it.  Was there anything you did like about the study? If yes: Can you tell us about this and why you liked this.  Since the study began have you felt differently about coming to school? Do you enjoy it less or more?  Do you feel that girls in your form have changed at all because they have been part of the study?  Do you feel that other people’s attitudes towards girls in the study have changed because they were In the study? | Probe to find out level of agreement. Were these key issues for many or just the odd person.  Probe to find out level of agreement. Were these key issues for many or just the odd person.  Explore the reasons why  Probe to find out what changes have occurred and why they think it resulted from being in this study.  Probe to find out who/what sort of people. Check family / friends/boyfriends/older men / boda boda…anyone else?  For each group find out how their attitudes have changed towards the girls and why this was because the girls were in the study. |
| --- | --- | --- |
| This section is on schooling and the study. One of the key outcomes for the study is school drop-out and sexual behaviours so here we will focus on whether this study might have helped prevent any drop-out.  For any question where they don’t have much to say then move on rather than try and force them to give a response. Concentrate on questions that they can give good information on. | Tell us about school drop out…have many girls who enrolled into secondary school in your year given up on schooling? Why did they leave school?  Thinking back, have you noticed any differences in the number of girls who drop out in the early forms – 1 & 2, compared to later form3 3 or 4?  Do you think girls who drop out of school are very different to girls like you who stay until form 4?  Do you think being in this study has made any difference to girls of your age, staying in school….or leaving school?  (If not mentioned) Do they think having a cup has made any difference or not to whether girls decide to drop out of school? | If yes – find out how many / what were key reasons for drop-out if known. (probe pregnancy/fees/peer pressure/absence etc) What can be done to prevent this drop-out?  Probe to get an explanation if they say yes – how and why. (If they haven’t noticed anything then move on) Are there differences in the reasons for drop-out over time?  Probe to get an explanation of their answers as to why (if any difference is identified)  Probe to get an explanation of their answers as to why (if any difference is identified) Were they to do with having a cup or was it the education part or soap? Or was it the whole package?  If yes: find out how and why, and the extent (many or few girls) |
| Now move to find out about school fees, attendance and performance, and whether being in the study might have some effect on these. We already know quite a lot about these so this is just to allow comparison across the different study arms – and also any changes in this group due to being in the study  For all questions that you follow up, find out if this is the opinion of most of the group or just one or two opinions. Consider asking ‘does anyone agree or disagree with this’  Also find out if many or just a few girls are affected. | We have seen that girls are sent home for fees, how long is it usually before they are allowed back into school? Are they able to continue as normal or does this affect their schooling in any way?  Do many girls have to repeat the form again?  Is there anything that makes it difficult for girls like yourselves to work in class and do well in your studies?  Thinking back to when you first started in the study, has anything changed since then because of being in the study which makes it easier or more difficult for you to work in class and do well in your studies?  Has being given a menstrual cup made any difference to girls ability to work in class and study well?  Moving on to school absence - Do many girls in your year miss school often?  Thinking back to when you started being in the study, do you think there have been any changes in girls’ ability to attend school as a result of being in the study?  Has being given a menstrual cup made any difference in girls ability to attend school?  Tell us about your relationships with the teachers at your school – are they supportive of schoolgirls like yourselves?  So, again thinking back to when the study started, have teachers attitudes or behaviour towards you changed at all or has it remained pretty much the same? | Find out how long they tend to be away from school, whether they return, how they catch up and whether this has any effect on them in the short or long term.  If this is common find out why they have to and how it affects these girls. Find out if teachers support these girls or whether they make things more difficult.  Find out what, if anything does and why.  If they identify anything then find out what this is and why it has changed as a result of the study  Were they to do with having a cup or was it the education part or soap? Or was it the whole package?  If so, find out why, and the extent to which it has made a difference. (A lot / little..to many / few girls)  If so, find out whether many or just a few girls miss school and what the key reasons are. Do not push them to talk about menstruation – let them give the different reasons  Find out what these changes were, why they happened i.e how they were due to the study, when did they happen (was this a gradual/sudden change, over the last few months or at the start of the study etc) Were they to do with having a cup or was it the education part or soap? Or was it the whole package?  If so, find out why, and the extent to which it has made a difference. (A lot / little..to many / few girls)  If not, explore why girls think this  If changes, then explore how and why |
| This section of the study is to find out if being in the study has made any difference to how girls feel about themselves (e.g. self-esteem and confidence)  Let them bring up any mention of the menstrual cup making a difference or not. Only ask as a last resort if they haven’t already mentioned | Do girls in your year feel shy or confident when they are in school? Why is this?  What about being around boys in your year….Do you think girls in your year are confident or shy in dealing with these boys? Why is this?  Do you think there have been any changes in girls confidence in the 2 years or so since being in the study? Do you think these changes are a result of being in the study?  (If not already mentioned) Do you think having a menstrual cup has made any difference or not in girls confidence generally? | Find out if girls feel able to say ‘no’ and stand up to boys, whether boys can pressure or ‘bully’ them, or whether it is the other way around and girls can make boys do what they want. If so, how do they manage this?  If they suggest any changes are because of the study then find out what these are and why they have happened. Were they to do with having a cup or was it the education part or soap? Or was it the whole package?  If yes: find out how and why, and the extent (A lot / little..to many / few girls) |
| We also want to know if being in the study has made any difference in girls relationships with boys or men, particularly in relation to having sex for money or gifts.  Some of this information may have already come out from the questions above, so just ask and probe once.  Again, let them bring up any mention of the menstrual cup making a difference or not. Only mention as a last question if they haven’t | Do girls in your year have relationships with boys or men? Tell us what sort of relationships they are….  *If they describe sexual relationships*, ask: we have heard that some girls have sex for gifts or money, do you think this happens to other girls in your forms (in your school, or other schools you know of) ?  Which schoolgirls – all or just some? What makes some girls need to do this but not others? What are the differences between these girls?  Do you think that relationships girls in the study have with boys / men has changed at all because of the study?  (If not discussed above) Do you think that having a cup has had any impact or not on girls’ relationships with boys / men? | Probe to find out if they are just friendships, sexual or a mix. Are they mostly with boys of their own age, or with older men. Do girls have multiple relationships, or serial (i.e they move on from one straight to the next). Do girls really want these relationships or are they pressured (if so, why and how)  Probe to find out what they receive, gifts or money –if cash what do they use the money for**** (NB this is important)  Probe to find out if girls think this is a good situation – are they in control of the boys and it is their choice, or do they do this out of real need / desperation. Is it common to have many different boyfriends or do they stick to just one? Do they want this situation to change? If yes: how could it be changed?  If yes, probe to find out in what way they have changed, how this relates to being in the study (if at all), and when these changes occurred, and how quickly or gradual they were. Were they to do with having a cup or was it the education part or soap? Or was it the whole package?  If not /not really just move on  Do the girls think the cup has stopped, or reduced any sexual transactions for money for helping deal with their menstruation  In addition, when girls consider yes, do the girls still need to have sex with boys and men for money for other things – what things - probe  If it has made a difference, explore what type of relationship – friendship / sexual / sex for money – how and why this has made a difference. whether this is to many girls / whether it has stopped all sex for money or just reduced the number of times this happens or the number of boyfriends needed etc |
| Questions on cash which will be useful for comparing with girls in the other arms of the study.  Only ask these questions if they haven’t already been covered. | Are girls like yourselves able to get and save money?  Have your needs for money changed at all since being given a menstrual cup?  What sort of things do girls in your year need money for?  If you received 500/ a month, what would you use it for? | If yes, find out how they get money from and what they use it for, or if they save - what is this saved for?  If yes, how and in what way? What financial impact has having a cup made to these girls. If they need less money now has this changed their lives at all? (probe chores/homework/boda boda)  Find out if they think this would be enough for their needs, and if not, why not  . |
| Questions on menstruation which will be useful for comparing with girls in other arms of the study. Also bear in mind that girls in the cup group will have received puberty & hygiene training and some soap as well as a cup, so there may have been some changes or difference expectations because of the study already.  As before only ask these questions if these haven’t already been covered | Are there any other programmes in your community that give pads or money to families to help girls  Does your school give sanitary pads to girls?  What are girls in your year using to manage your periods? Are girls using the menstrual cup? | If yes, find out more – DREAMS or others – in your form are there many girls supported with this? how many? How does it help them? Do you think it makes any difference to their schooling? What about their lives generally?  If yes, find out more - Is this to all girls, or just if they need one in an emergency? If to all girls – how many do they give out? How often? Are they enough? What are the pads like? What is good about this? What is not good about this?  Find out what they think of these materials.  If they are not using the cup find out why and what they use instead, what did they do with their cup? If using pads, how do they afford them?  If they use the cup probe to find out what they think about it – any likes or dislikes / challenges. What, if any, impact has it had on their lives? Would they recommend it to other girls / women? |
|  |  |  |

**GIRLS – CASH GROUP ENDLINE FGD**

Information / Instructions Key Topics / Questions Probes

| So this is just to make sure they are really aware and not talking about a different intervention. Make sure they clearly understand that it is our study being discussed and not another intervention that is happening in school or community. These girls will have received puberty hygiene education, bloods taken for HIV and survey participation. They will also have received the cash transfer card so it is likely that they will discuss this as part of liking/ not liking the study. If they bring this up themselves then dig deep here and find out all the cash relevant information now rather than later. If they do not mention it, then wait until the section specifically on the cash to ask them.  Also be aware that they might not have noticed much or have much of an opinion so don’t force them to come up with answers if they can’t really think of anything.  Get key responses, find out level of agreement generally but don’t go round the room so that everyone has to say something. Just draw out if shy and look like they want to contribute but louder girls are overpowering them.  If girls mention anything that we would have asked about later in this FGD, you can dig in here and gather all the information then miss out later, or ask later – but you don’t need to ask twice.  In each section dont force girls to talk about menstruation issues (OK if it comes up spontaneously) - there is a section on menstruation where it can be discussed | Lets talk about the KEMRI study first. What did you think about being in the study?  Was there anything you didn’t like about the study? If yes; can you tell us about this and why you didn’t like it.  Was there anything you did like about the study? If yes: Can you tell us about this and why you liked this.  Since the study began have you felt differently about coming to school? Do you enjoy it less or more?  Do you feel that girls in your form have changed at all because they have been part of the study?  Do you feel that other people’s attitudes towards girls in the study have changed because they were In the study? | Probe to find out level of agreement. Were these key issues for many or just the odd person.  Probe to find out level of agreement. Were these key issues for many or just the odd person.  Explore the reasons why  Probe to find out what changes have occurred and why they think it resulted from being in this study.  Probe to find out who/what sort of people. Check family / friends/boyfriends/older men / boda boda…anyone else?  For each group find out how their attitudes have changed towards the girls and why this was because the girls were in the study. |
| --- | --- | --- |
| This section is on schooling and the study. One of the key outcomes for the study is school drop-out and sexual behaviours so here we will focus on whether this study and having cash to spend might have helped prevent any drop-out.  For any question where they don’t have much to say then move on rather than try and force them to give a response. Concentrate on questions that they can give good information on. | Tell us about school drop out…have many girls who enrolled into secondary school in your year given up on schooling? Why did they leave school?  Thinking back, have you noticed any differences in the number of girls who drop out in the early forms – 1 & 2, compared to later form 3 or 4?  Do you think girls who drop out of school are very different to girls like you who stay until form 4?  Do you think being in this study has made any difference to girls of your age, staying in school….or leaving school?  (If not mentioned) Do you think that having the cash transfer has made any difference or not to whether girls decide to drop out of school? | If yes – find out how many / what were key reasons for drop-out if known. (probe pregnancy/fees/peer pressure/absence etc) What can be done to prevent this drop-out?  Probe to get an explanation if they say yes – how and why. (If they haven’t noticed anything then move on) Are there differences in the reasons for drop-out over time?  Probe to get an explanation of their answers as to why (if any difference is identified)  Probe to get an explanation of their answers as to why (if any difference is identified) Were they to do with having cash (so what specifically was it spent on that made the difference to schooling) or was it the education part or soap? Or was it the whole package?  If yes: find out how and why, and the extent (many or few girls). Probe specifically to find out how having the cash transfer meant girls did / did not drop out – what was the money spent on that made the difference. |
| Now move to find out about school fees, attendance and performance, and whether being in the study might have some effect on these. We already know quite a lot about these so this is just to allow comparison across the different study arms – and also any changes in this group due to being in the study  For all questions that you follow up, find out if this is the opinion of most of the group or just one or two opinions. Consider asking ‘does anyone agree or disagree with this’  Also find out if many or just a few girls are affected. | We have seen that girls are sent home for fees, how long is it usually before they are allowed back into school? Are they able to continue as normal or does this affect their schooling in any way?  Do many girls have to repeat the form again?  Is there anything that makes it difficult for girls like yourselves to work in class and do well in your studies?  Thinking back to when you first started in the study, has anything changed since then because of being in the study which makes it easier or more difficult for you to work in class and do well in your studies?  Has being given cash transfer/pocketmoney made any difference to girls ability to work in class and study well?  Moving on to school absence - Do many girls in your year miss school often?  Thinking back to when you started being in the study, do you think there have been any changes in girls’ ability to attend school as a result of being in the study?  Has being given cash transfer/po made any difference in girls ability to attend school? | Find out how long they tend to be away from school, whether they return, how they catch up and whether this has any effect on them in the short or long term.  If not already discussed, find out if having cash transfer meant that some of the money went towards paying fees. If so, did this make much or any difference to whether girls were sent home for it and missed much school.  How many – why?  If this is common find out why they have to and how it affects these girls. Find out if teachers support these girls or whether they make things more difficult.  Find out what, if anything does and why.  If they identify anything then find out what this is and why it has changed as a result of the study  Were they to do with having cash transfer or was it the education part or soap? Or was it the whole package?  If so, find out why, and the extent to which it has made a difference. (A lot / little..to many / few girls)  If so, find out whether many or just a few girls miss school and what the key reasons are. Do not push them to talk about menstruation – let them give the different reasons  Find out what these changes were, why they happened i.e how they were due to the study, when did they happen (was this a gradual/sudden change, over the last few months or at the start of the study etc) Were they to do with having cash transfer or was it the education part or soap? Or was it the whole package?  If so, find out why, and the extent to which it has made a difference. (A lot / little..to many / few girls) |
| This section of the study is to find out if being in the study has made any difference to how girls feel about themselves (e.g. self-esteem and confidence)  Let them bring up any mention of the cash transfer making a difference or not. Only ask as a last resort if they haven’t already mentioned | Do girls in your year feel shy or confident when they are in school? Why is this?  What about being around boys in your year….Do you think girls in your year are confident or shy in dealing with these boys? Why is this?  Do you think there have been any changes in girls confidence in the 2 years or so since being in the study? Do you think these changes are a result of being in the study?  (If not already mentioned) Do you think having cash has made any difference or not in girls confidence generally? | Find out if girls feel able to say ‘no’ and stand up to boys, whether boys can pressure or ‘bully’ them, or whether it is the other way around and girls can make boys do what they want. If so, how do they manage this?  If they suggest any changes are because of the study then find out what these are and why they have happened. Were they to do with having cash or was it the education part or soap? Or was it the whole package?  If yes: find out how and why, and the extent (A lot / little..to many / few girls) |
| We also want to know if being in the study has made any difference in girls relationships with boys or men, particularly in relation to having sex for money or gifts.  Some of this information may have already come out from the questions above, so just ask and probe once.  Again, let them bring up any mention of cash transfer making a difference or not. Only mention as a last question if they haven’t | Do girls in your year have relationships with boys or men? Tell us what sort of relationships they are….  *If they describe sexual relationships*, ask: we have heard that some girls have sex for gifts or money, do you think this happens to other girls in your forms (in your school, or other schools you know of) ?  Which schoolgirls – all or just some? What makes some girls need to do this but not others? What are the differences between these girls?  Do you think that relationships girls in the study have with boys / men has changed at all because of the study?  (If not discussed above) Do you think that having cash transfer/pocket money has had any impact or not on girls’ relationships with boys / men? | Probe to find out if they are just friendships, sexual or a mix. Are they mostly with boys of their own age, or with older men. Do girls have multiple relationships, or serial (i.e they move on from one straight to the next). Do girls really want these relationships or are they pressured (if so, why and how)  Probe to find out what they receive, gifts or money –if cash what do they use the money for**** (NB this is important)  Probe to find out if girls think this is a good situation – are they in control of the boys and it is their choice, or do they do this out of real need / desperation. Is it common to have many different boyfriends or do they stick to just one? Do they want this situation to change? If yes: how could it be changed?  If yes, probe to find out in what way they have changed, how this relates to being in the study (if at all), and when these changes occurred, and how quickly or gradual they were. Were they to do with having cash or was it the education part or soap? Or was it the whole package?  If not /not really just move on  If it has made a difference, explore what type of relationship it has changed and why– friendship / sexual / sex for money.  Do the girls think that having their own money has stopped having sex for money altogether, or reduced the number of sexual transactions for money, or the number of boyfriends needed.  If it has reduced but not stopped find out what sort of things do girls still need or want money for? |
| Questions on cash which will be useful for comparing with girls in the other arms of the study.  Only ask these questions if they haven’t already been covered. | What do you use the money from the study for?  As well as the money you receive from the study, do girls like yourselves earn or obtain money from any other source?  Has this study made any difference at all to your lives? | Find out if they spend – what on – do they have to give any away / who to / why?  Are they able to save any – what for?  Do family members often take the money from girls– who, why – what do you think is the consequence of this?  If yes, find out how they get money from and what they use it for. Why do they need this extra money  If yes, how and in what way? (probe chores/homework/boda boda)  . |
| Questions on menstruation which will be useful for comparing with girls in other arms of the study. Also bear in mind that girls in the cash transfer group will have received puberty & hygiene training as well as cash transfer, so there may have been some changes or difference expectations because of the study already.  As before only ask these questions if these haven’t already been covered. Make sure that you have asked (or it has been answered) if they use cash for buying pads or still need / get from parent, boyfriends or through transactional sex | Are there any other programmes in your community that give pads or money to families to help girls  Does your school give sanitary pads to girls?  What are girls in your year using to manage their periods?  Has this changed in the years since starting this study?  Do girls like yourselves find menstruation easy to manage now? Why / why not? What would help?  Have any of you heard of the menstrual cup? | If yes, find out more – DREAMS or others – in your form are there many girls supported with this? how many? How does it help them? Do you think it makes any difference to their schooling? What about their lives generally?  If yes, find out more - Is this to all girls, or just if they need one in an emergency? If to all girls – how many do they give out? How often? Are they enough? What are the pads like? What is good about this? What is not good about this?  Find out what they think of these materials. If not already mentioned check if girls are using their cash transfer money for sanitary needs.  Find out what any changes are and why they have changed. – NB**** probe did they use cash for buying pads or still need or get from parents or boyfriends  If yes, find out what they know about it and what they think. |
|  |  |  |

**GIRLS CASH+CUP GROUP ENDLINE FGD**

Information / Instructions Key Topics / Questions Probes

| So this is just to make sure they are really aware and not talking about a different intervention. Make sure they clearly understand that it is our study being discussed and not another intervention that is happening in school or community. These girls will have received puberty hygiene education, hand soap, bloods taken for HIV and survey participation. They will also have received the cash transfer card and received a menstrual cup so it is likely that they will discuss any of these as part of liking/ not liking the study. If they bring any of these things up themselves then dig deep here and find out all the cash or cup relevant information now rather than later. If they do not mention it, then wait until the section specifically on the cash or cup to ask them  Also be aware that they might not have noticed much or have much of an opinion so don’t force them to come up with answers if they can’t really think of anything.  Get key responses, find out level of agreement generally but don’t go round the room so that everyone has to say something. Just draw out if shy and look like they want to contribute but louder girls are overpowering them.  If girls mention anything that we would have asked about later in this FGD, you can dig in here and gather all the information then miss out later, or ask later – but you don’t need to ask twice. | Lets talk about the KEMRI study first. What did you think about being in the study?  Was there anything you didn’t like about the study? If yes; can you tell us about this and why you didn’t like it.  Was there anything you did like about the study? If yes: Can you tell us about this and why you liked this.  Since the study began have you felt differently about coming to school? Do you enjoy it less or more?  Do you feel that girls in your form have changed at all because they have been part of the study?  Do you feel that other people’s attitudes towards girls in the study have changed because they were in the study? | Probe to find out level of agreement. Were these key issues for many or just the odd person.  Probe to find out level of agreement. Were these key issues for many or just the odd person.  Explore the reasons why  Probe to find out what changes have occurred and why they think it resulted from being in this study.  Probe to find out who/what sort of people. Check family / friends/boyfriends/older men / boda boda…anyone else?  For each group find out how their attitudes have changed towards the girls and why this was because the girls were in the study. |
| --- | --- | --- |
| This section is on schooling and the study. One of the key outcomes for the study is school drop-out and sexual behaviours so here we will focus on whether this study and having cash to spend or a cup might have helped prevent any drop-out.  For any question where they don’t have much to say then move on rather than try and force them to give a response. Concentrate on questions that they can give good information on. | Tell us about school drop out…have many girls who enrolled into secondary school in your year given up on schooling? Why did they leave school?  Thinking back, have you noticed any differences in the number of girls who drop out in the early forms – 1 & 2, compared to later form 3 or 4?  Do you think girls who drop out of school are very different to girls like you who stay until form 4?  Do you think being in this study has made any difference to girls of your age, staying in school….or leaving school?  (If not mentioned) Do you think that having the cash transfer and the menstrual cup has made any difference or not to whether girls decide to drop out of school?  (If any difference identified) Do you think the cash or the cup was most important in making a difference to girls dropping out of school, or was it a combination of having both? | If yes – find out how many / what were key reasons for drop-out if known. (probe pregnancy/fees/peer pressure/absence etc) What can be done to prevent this drop-out?  Probe to get an explanation if they say yes – how and why. (If they haven’t noticed anything then move on) Are there differences in the reasons for drop-out over time?  Probe to get an explanation of their answers as to why (if any difference is identified)  Probe to get an explanation of their answers as to why (if any difference is identified) Were they to do with having cash (so what specifically was it spent on that made the difference to schooling), or having a cup or was it the education part or soap? Or was it the whole package?  If yes: find out how and why, and the extent (many or few girls). Probe specifically to find out how having the cash transfer meant girls did / did not drop out – what was the money spent on that made the difference. Then probe on the cup  Ask them to explain their responses |
| Now move to find out about school fees, attendance and performance, and whether being in the study might have some effect on these. We already know quite a lot about these so this is just to allow comparison across the different study arms – and also any changes in this group due to being in the study  For all questions that you follow up, find out if this is the opinion of most of the group or just one or two opinions. Consider asking ‘does anyone agree or disagree with this’  Also find out if many or just a few girls are affected. | We have seen that girls are sent home for fees, how long is it usually before they are allowed back into school? Are they able to continue as normal or does this affect their schooling in any way?  Do many girls have to repeat the form again?  Is there anything that makes it difficult for girls like yourselves to work in class and do well in your studies?  Thinking back to when you first started in the study, has anything changed since then because of being in the study which makes it easier or more difficult for you to work in class and do well in your studies?  If not mentioned) Do you think that having the cash transfer and the menstrual cup has made any difference or not to girls ability to work in class and do well in your studies  (If any difference identified) Do you think the cash or the cup was most important in making a difference to girls dropping out of school, or was it a combination of having both?  Moving on to school absence - Do many girls in your year miss school often?  Thinking back to when you started being in the study, do you think there have been any changes in girls’ ability to attend school as a result of being in the study?  If not mentioned) Do you think that having the cash transfer and the menstrual cup has made any difference or not to girls ability to attend school  (If any difference identified) Do you think the cash or the cup was most important in making a difference to girls dropping out of school, or was it a combination of having both | Find out how long they tend to be away from school, whether they return, how they catch up and whether this has any effect on them in the short or long term.  If not already discussed, find out if having cash transfer meant that some of the money went towards paying fees. If so, did this make much or any difference to whether girls were sent home for it and missed much school.  If this is common find out why they have to and how it affects these girls. Find out if teachers support these girls or whether they make things more difficult.  Find out what, if anything does and why.  If they identify anything then find out what this is and why it has changed as a result of the study.  Were they to do with having cash transfer or the cup or was it the education part or soap? Or was it the whole package?  Probe to get an explanation of their answers as to why (if any difference is identified) Were they to do with having cash (so what specifically was it spent on that made the difference to schooling), or having a cup or was it the education part or soap? Or was it the whole package?  If yes: find out how and why, and the extent (many or few girls). Probe specifically to find out how having the cash transfer meant girls did / did do well in their studies – what was the money spent on that made the difference. Then probe on the cup  Ask them to explain their responses  If so, find out whether many or just a few girls miss school and what the key reasons are. Do not push them to talk about menstruation – let them give the different reasons  Find out what these changes were, why they happened i.e how they were due to the study, when did they happen (was this a gradual/sudden change, over the last few months or at the start of the study etc) Were they to do with having cash transfer, a menstrual cup or was it the education part or soap? Or was it the whole package?  If yes: find out how and why, and the extent (many or few girls). Probe specifically to find out how having the cash transfer meant girls could attend more – what was the money spent on that made the difference. Then probe on the cup  Ask them to explain the reason for their answers |
| This section of the study is to find out if being in the study has made any difference to how girls feel about themselves (e.g. self-esteem and confidence)  Let them bring up any mention of the cash transfer or menstrual cup making a difference or not. Only ask as a last resort if they haven’t already mentioned | Do girls in your year feel shy or confident when they are in school? Why is this?  What about being around boys in your year….Do you think girls in your year are confident or shy in dealing with these boys? Why is this?  Do you think there have been any changes in girls confidence in the 2 years or so since being in the study? Do you think these changes are a result of being in the study?  (If not already mentioned) If not mentioned) Do you think that having the cash transfer and the menstrual cup has made any difference or not to girls confidence?  Do you think the cash or the cup was most important in making a difference to girls confidence, or was it a combination of having both? | Find out if girls feel able to say ‘no’ and stand up to boys, whether boys can pressure or ‘bully’ them, or whether it is the other way around and girls can make boys do what they want. If so, how do they manage this?  If they suggest any changes are because of the study then find out what these are and why they have happened. Were they to do with having cash, a menstrual cup or was it the education part or soap? Or was it the whole package?  If yes: find out how and why, and the extent (many or few girls). Probe specifically to find out how having the cash transfer meant girls could attend more – what was the money spent on that made the difference. Then probe on the cup  Ask them to explain the reason for their answers |
| We also want to know if being in the study has made any difference in girls relationships with boys or men, particularly in relation to having sex for money or gifts.  Some of this information may have already come out from the questions above, so just ask and probe once.  Again, let them bring up any mention of cash transfer or a menstrual cup making a difference or not. Only mention as a last question if they haven’t | Do girls in your year have relationships with boys or men? Tell us what sort of relationships they are….  *If they describe sexual relationships*, ask: we have heard that some girls have sex for gifts or money, do you think this happens to other girls in your forms (in your school, or other schools you know of) ?  Which schoolgirls – all or just some? What makes some girls need to do this but not others? What are the differences between these girls?  Do you think that relationships girls in the study have with boys / men has changed at all because of the study?  (If not already mentioned) Do you think that having the cash transfer and the menstrual cup has made any difference or not to girls relationships with boys/men  (If any difference identified) Do you think the cash or the cup was most important in making a difference in girls relationships with boys / men , or was it a combination of having both? | Probe to find out if they are just friendships, sexual or a mix. Are they mostly with boys of their own age, or with older men. Do girls have multiple relationships, or serial (i.e they move on from one straight to the next). Do girls really want these relationships or are they pressured (if so, why and how)  Probe to find out what they receive, gifts or money –if cash what do they use the money for**** (NB this is important)  Probe to find out if girls think this is a good situation – are they in control of the boys and it is their choice, or do they do this out of real need / desperation. Is it common to have many different boyfriends or do they stick to just one? Do they want this situation to change? If yes: how could it be changed?  If yes, probe to find out in what way they have changed, how this relates to being in the study (if at all), and when these changes occurred, and how quickly or gradual they were.  Explore what type of relationship it has changed and why– friendship / sexual / sex for money.  Do the girls think that having their own money or a cup has stopped having sex for money altogether, or reduced the number of sexual transactions for money, or the number of boyfriends needed.  Were changes to do with having cash or was it the cup, or even the education part -? Or was it the whole package?  If not /not really just move on  If yes: find out how and why, and the extent (many or few girls). Probe specifically to find out how having the cash transfer meant girls relationships changed– what was the money spent on that made the difference. Then probe on the cup  Ask them to explain the reason for their answers |
| Questions on cash which will be useful for comparing with girls in the other arms of the study.  Only ask these questions if they haven’t already been covered. | What do you use the money from the study for?  As well as the money you receive from the study, do girls like yourselves earn or obtain money from any other source? | Find out if they spend – what on – do they have to give any away / who to / why?  Are they able to save any – what for?  Do family members often take the money – who, why – what do you think is the consequence of this?  If yes, find out how they get money from and what they use it for. Why do they need this extra money  . |
| Questions on menstruation which will be useful for comparing with girls in other arms of the study. Also bear in mind that girls in the cash transfer group will have received puberty & hygiene training as well as cash transfer and a cup, so there may have been some changes or difference expectations because of the study already.  As before only ask these questions if these haven’t already been covered | Are there any other programmes in your community that give pads or money to families to help girls  Does your school give sanitary pads to girls?  What are girls in your year using to manage their periods? Are girls using the menstrual cup? | If yes, find out more – DREAMS or others – in your form are there many girls supported with this? how many? How does it help them? Do you think it makes any difference to their schooling? What about their lives generally?  If yes, find out more - Is this to all girls, or just if they need one in an emergency? If to all girls – how many do they give out? How often? Are they enough? What are the pads like? What is good about this? What is not good about this?  Find out what they think of these materials.  If they are not using the cup find out why and what they use instead, what did they do with their cup? If using pads, how do they afford them?  If they use the cup probe to find out what they think about it – any likes or dislikes / challenges. What, if any, impact has it had on their lives? Would they recommend it to other girls / women? |
|  |  |  |
