# Supplementary material for: ‘You don’t have to sleep with a man to get how to survive’: Girl’s perceptions of an intervention study aimed at improving sexual and reproductive health and schooling outcomes
Source: PLOS Glob Public Health. 2022 Oct 13;2(10):e0000987. doi: 10.1371/journal.pgph.0000987 (PMC10021241; doi:10.1371/journal.pgph.0000987)
Supplement: S3 Table — (DOCX) [file pgph.0000987.s005.docx]

**MIDLINE FGD SCHOOLGIRLS CONTROL GROUP**

| **MODERATOR:** Document required information as appropriate for each FGD using the formats provided below. Date: _____/_______/________  Initials: Moderator: ______ Note Taker______ Recorder Number: ____ Folder/File Name (location on recorder): _______________________  Interview location (Venue): _________________________________________________________  FGD Group: _____________________________________________________________________ FGD Number: _____  Time Start: _______________ Time stop: ________________ No. Participants at start of FGD: ________ No. Participants at the end of FGD: _________  **Demographic information for every FGD participant *[to be completed on a one-to-one basis, immediately after consent is obtained]***   \| **Participant number or**  **Fake name** \| **Age in completed years** \| **Ethnic group** \| \| --- \| --- \| --- \| \| 1 \|  \|  \| \| 2 \|  \|  \| \| 3 \|  \|  \| \| 4 \|  \|  \| \| 5 \|  \|  \| \| 6 \|  \|  \| \| 7 \|  \|  \| \| 8 \|  \|  \| \| 9 \|  \|  \| \| 10 \|  \|  \| \| 11 \|  \|  \| \| 12 \|  \|  \|   **COMMENTS – reasons for withdrawal, refusal, ambience of FG, level of interest, disagreements, etc** |
| --- | --- | --- | --- | --- | --- | --- | --- | --- | --- | --- | --- | --- | --- | --- | --- | --- | --- | --- | --- | --- | --- | --- | --- | --- | --- | --- | --- | --- | --- | --- | --- | --- | --- | --- | --- | --- | --- | --- | --- |
| **Introduction**  Thank you so much for your willingness to take part in this group discussion. My name is **[Name]**. I am from the KEMRI. We are doing a research study  to see if we can help prevent girls from dropping out of school early  and keep them healthy as well.  We’re interested in hearing from you about what it is like to be a schoolgirl, at **[Name of community/school]** and whether being part of the study has made any difference to you and your peers.  Often people from outside think they know what you think regarding these issues when they really don’t. To us, you are the real experts, and there’s a lot we can learn from you.  So today we would like to hear your views . This is very informal; you can talk about anything you think is important for us to know. I also want to remind you that everything we talk about today is confidential.  No one will hear this tape except for people working on the project. Whenever we write a report, we will use numbers or fake names so no one can identify you.  If there are any questions you’d rather not answer, just let me know - that’s fine.  Your frank responses and discussion will be most helpful to us as we try to really understand these issues. Remember, your answers to our questions will not be considered “right” or “wrong”, because we want to know about what people think. They are merely information you will provide based on your experiences, observations, or feelings. Everyone’s views are equally important. It’s fine to disagree with other people’s views, but if you do, it’s important to disagree in a respectful and polite manner. It’s important for you to talk in turns to speak, because if you all speak at once, we will not have a clear recording. If you disagree with something anyone says, you can say ‘I disagree’ and then wait for them to finish before you speak.  **Explain the role of note-takers and tape-recorder**  **Give a few minutes for answering any questions regarding the FGD**  Please note the questions here:  ______________________________________________________________________________________________________________________________  ______________________________________________________________________________________________________________________________  ______________________________________________________________________________________________________________________________  ______________________________________________________________________________________________________________________________  ______________________________________________________________________________________________________________________________ |

| Theme |  |  |
| --- | --- | --- |
| **Schooling**  Here we are trying to find out what girls hope to achieve by being at school and what the barriers to being educated are. We need to understand how we can help keep them in school. | **What have you liked about the study so far?**  **What have you not liked about the study?**  **Is there anything that has made it difficult for you, or girls like yourself, to remain at school?** (find out what this is, why it is a problem and what they think would help)  **What are the reasons that girls drop-out from school?** (If they discuss menstrual issues or money difficulties then probe the items in the sections on menstrual issues / Cash)  **What could help girls to stay in school?**  **Do you find it much different being in form 3 compared to last year in form 2?** (probe why so)  **Is there more pressure on you now that you are in form 3?** (probe to find what the pressures are – coursework / homework / family / friends / chores / relationships etc and how they affect the girls) | |
| **School attendance and performance**  Here we want to find out if there have been any changes in drop out, absenteeism and performance since the study started. NB be aware that we know some of this already from previous FGDs so here the emphasis is on finding if there are any differences since the trial started and if so, why these have happened. | **Since the study started have there been any changes in girls dropping out from school?** (get them to describe any so that we understand why these have happened)  **What about changes in girls being absent from school** (get them to describe any so that we understand why these have happened)  **Have girls ability to work in class, and to do well in lessons changed since they have started the study** (Get them to describe any changes so that we understand why these have happened)  **What makes it difficult for girls to do well in school**? (find out why and what can be done to improve things for girls) | |
| **Cash**  So here we want to know if lack of money prevents girls from being at school or doing well at school, and whether this has changed at all since the study started. | **… Do you think you and your peers have enough money for what you need?** (get them to explain their responses so that we know what they need, why they need it – and if they have enough money, then where it comes from)  **Has this changed since taking part in the study?** (find out why / why not)  **Do girls notice any difference with their family finances since being part of the study?** (if yes, get them to explain why and what difference this makes)  **Does lack of money make it difficult for girls to stay at school**? (find out about the circumstances these difficulties arise, and what they think might help) **Has this changed since being part of the study?** (find out why / why not)  **Does lack of money make it difficult for girls to do well at school**? (find out why and what might help) **Has this changed since being part of the study**? (find out why / why not) | |
| **Menstrual Issues**  We already know a lot about this so just concentrate on whether the schools are giving pads out – and if so how useful this is. We need to know if this is a good option instead of giving cups or cash.  Also check whether there have been any change in menstrual circumstances since the study began, and how useful the hygiene and puberty training was. | **Since being in the study has this made a difference to girls menstrual issues?** (Probe for any differences and why they have occurred)  **Does your school give pads to girls?** (If so, find out the circumstances e.g how they do this, whether it is to all or some girls, on regular basis or only if they begin menstruating without having any protection etc and how helpful this is, what is good and what is not good about this)  If not: **What do you think about schools giving out pads to girls?** (find out what the girls think of this option and why))  **Thinking back to the hygiene and puberty training that you received at the start of the study, what was your opinion of it and why? (**explore if any additional information would have been useful / why or if the information provided differently | |
| **Relationships**  We want to find out whether and how these affect girls ability to stay in school and / or do well at school. We are particularly interested whether girls might stop having transactional sex, or have fewer partners, if they were provided with enough resources (Cash / menstrual protection) | **Do you think being part of the study has made any difference to girls relationships with boys / men?** (find out what specific behaviours / what differences / what has caused them. Remember girls received puberty / hygiene training / soap / cup  **Since being in the study do girls still need to have ‘boyfriends’ to buy other essential items …?** (find out what their reasons are and what sort of things they buy. Do they have one partner for this or many / has this changed since being in the study) | |
|  |  |  |

**MIDLINE FGD SCHOOLGIRLS CUP GROUP**

| Theme |  |  |
| --- | --- | --- |
| **Schooling**  Here we are trying to find out what girls hope to achieve by being at school and what the barriers to being educated are. We need to understand how we can help keep them in school. | **What have you liked about the study so far?**  **What have you not liked about the study?**  **Is there anything that has made it difficult for you, or girls like yourself, to remain at school?** (find out what this is, why it is a problem and what they think would help)  **What are the reasons that girls drop-out from school?** (If they discuss menstrual issues or money difficulties then probe the items in the sections on menstrual issues / Cash)  **What could help girls to stay in school?**  **Do you find it much different being in form 3 compared to last year in form 2?** (probe why so)  **Is there more pressure on you now that you are in form 3?** (probe to find what the pressures are – coursework / homework / family / friends / chores / relationships etc and how they affect the girls) | |
| **School attendance and performance**  Here we want to find out if there have been any changes in drop out, absenteeism and performance since the girls were given a cup. NB be aware that we know some of this already from previous FGDs so here the emphasis is on finding if there are any differences since the trial started and if so, why these have happened. If they see no differences we need to know this, so don’t steer them to agreeing if they have other thoughts – instead probe on these | **Since the study started have there been any changes in girls dropping out from school?** (get them to describe any so that we understand why these have happened and whether this is because of getting a cup)  **What about changes in girls being absent from school** (get them to describe any so that we understand why these have happened)  **Have girls ability to work in class, and to do well in lessons changed since they have started the study** (Get them to describe any changes so that we understand why these have happened)  **What makes it difficult for girls to do well in school**? (find out why and what can be done to improve things for girls) | |
| **Experience of Using the Cup**  We need to find out their views and experiences of early cup use, and what impact it has, if any on their schooling. We also need to find out whether SWAP using men for training was an issue for the girls. | **Can you remember back to the training that you had before you were given the cup – what did they do? What did you think about t?** (find out if it was good enough or whether there needed to be more information or guidance (showing how), / something different – what was useful / not useful etc  **Were you taught by females or males?** If any men: **What did you think about this? (**Find out whether it had any effect on how they learned how to use the cup, why this happened and what effect it had)?  **What did you and your friends first think about the menstrual cup when you received it?** (find out if they and their friends tried using it straight away and what their experiences were. If they waited before they tried it, what did they wait for / how long / What made them eventually try it / Did girls help each other/ how?)  **What do you and your friends think about using the menstrual cup now?** (probe if girls use / why / not / any difficulties in using, if so, what are they? Is there anything that would help them to use/ Do they use for the whole time they are menstruating, if not / why not / when do they use or not.  **Tell us about your experiences in inserting the cup** (at first/ now / at school / at home / day or night. Probe whether there was / is anything difficult, and what they are /whether anything has helped to make this better)  **Tell us about your experiences in emptying the cup** (at first / now, at school / at home / day or night. Probe whether there were any difficulties and what they were /are, and whether anything has helped make this better or easier)  **Tell us about your experiences in re-inserting the cup (**to start with and now, at school / at home / day or night. Probe whether there were any difficulties and what they were /are, and whether anything has helped make this better or easier)  **Are there any times / circumstances when it is not good to use the cup?** (if yes, probe when and why – ask about during sport, and whether it is used during sex – if so, what happens)  **Are there times it is just easier still, to use pads, cloth etc than using cup?** (find out why and if this happens often and to many / few girls)  **Are there some tricks/experiences you can share with us, to help other girls start and get used to using cups?**  **Going back to when you were first given a cup - What were the initial reactions of your family and others to the cups?** (Probe how did you explain it to them (who knows, do you keep it a secret – from who? why)?  **What do they think about it now? –**  **Tell us about whether anyone has asked you to share, or tried to take your cup and what happened if so?**  **Has anyone sold their cup? (**Probe why, what the money was used for and what they use instead for menstruation**)** | |
| **Menstrual Pads**  We already know a lot about this so just concentrate on whether the schools are giving pads out – in addition to girls receiving the menstrual cup and if so how useful this is … for example, if girls get pads do they forget /not want to use the cup?. | **Does your school give pads to girls?** (If so, find out the circumstances e.g how they do this, whether it is to all or some girls, on regular basis or only if they begin menstruating without having any protection.  If they are given pads **- Do girls prefer to use the pads they are given, or to use the cup?** (Ask them to explain their answers; probe – do they use both, which one for which circumstances?) | |
| **Cash**  So here we want to know if lack of money prevents girls from being at school or doing well at school, and whether this has changed at all since receiving a cup | **… Do you think you and your peers have enough money for what you need?** (get them to explain their responses so that we know what they need, why they need it – and if they have enough money, then where it comes from)  **Has this changed since having a cup?** (find out why / why not)  **Do girls notice any difference with their family finances since being given a cup?** (if yes, get them to explain why and what difference this makes)  **Does lack of money make it difficult for girls to stay at school**? (find out about the circumstances these difficulties arise, and what they think might help) **Has this changed since having a cup?** (find out why / why not)  **Does lack of money make it difficult for girls to do well at school**? (find out why and what might help) **Has this changed since having a cup**? (find out why / why not) | |
| **Relationships**  We want to find out whether being in the study and having a cup affects girls relationships with boys and men. We are particularly interested in whether having a cup has made any difference in girls need for transactional sex or reduced the number of partners. | **Do you think being part of the study has made any difference to girls relationships with boys / men?** (find out what specific behaviours / what differences / what has caused them. Remember girls received puberty / hygiene training / soap / cup  **Since being in the study do girls still need to have ‘boyfriends’ to buy other essential items …?** (find out what their reasons are and what sort of items they buy. Do they have one partner for this or many – has this changed since being in the study?) | |
|  |  |  |

**MIDLINE FGD SCHOOLGIRLS CASH TRANSFER GROUP**

| **Schooling**  Here we are trying to find out what girls hope to achieve by being at school and what the barriers to being educated are. We need to understand how we can help keep them in school. | **What have you liked about the study so far?**  **What have you not liked about the study?**  **Is there anything that has made it difficult for you, or girls like yourself, to remain at school?** (find out what this is, why it is a problem and what they think would help)  **What are the reasons that girls drop-out from school?** (If they discuss menstrual issues or money difficulties then probe the items in the sections on menstrual issues / Cash)  **What could help girls to stay in school?**  **Do you find it much different being in form 3 compared to last year in form 2?** (probe why so)  **Is there more pressure on you now that you are in form 3?** (probe to find what the pressures are – coursework / homework / family / friends / chores / relationships etc and how they affect the girls) |
| --- | --- |
| **School attendance and performance**  Here we want to find out if there have been any changes in drop out, absenteeism and performance since the girls were given cash NB be aware that we know some of this already from previous FGDs so here the emphasis is on finding if there are any differences since the trial started and if so, why these have happened. Are they because of girls receiving pocket money? | **Since the study started have there been any changes in girls dropping out from school?** (get them to describe any so that we understand why these have happened and whether this is because they now have cash)  **What about changes in girls being absent from school** (get them to describe any so that we understand why these have happened)  **Have girls ability to work in class, and to do well in lessons changed since they have started the study** (Get them to describe any changes so that we understand why these have happened)  **What makes it difficult for girls to do well in school**? (find out why and what can be done to improve things for girls) |
| **Cash**  So here we want to know if being given money will help girls to remain at school or to do well at school, why and how this may occur. What are they using their money for? We also need to check if the training was good enough and if girls have any problems accessing their cash. | **Can you remember your training on cash transfer – can you tell us what was good about it? What was bad about it? How would you improve it?**  **Do girls have any problems getting their pocket money from the study?** (Find out what the circumstances are, and whether this is a common problem)  **Do girls have any problems with their pocket money card?** (Find out what the circumstances are, and whether this is a common problem  **Do girls put any of their pocket money towards paying school fees?** (If so, find out if this is their choice or if they are pressured to do so, whether all of their cash is put towards this or just a portion – what do they use any money left for)  **Has being given pocket money made any difference to girls lives generally?** (find out how and why / why not)  **Has it made any difference to the families of girls in the study** (find out how and why / why not)  **What support do you get from your family in coming to school?** (If they don’t then find out why)  **What sort of things do girls spend their pocket money on?**  **Do you think girls now have enough money for what they need?** (why / why not?) **What other things would they like to buy but don’t have enough money for**  **How do girls make a choice what to spend money on**? (get them to explain their responses so that we know what they need, why they need it)  **is it possible for any girls to able to save any pocket money?** (find out if this is easier than before they were in the study, and what they are saving for)  **is it possible for any girls to budget their pocket money? Does it last for the full term?**  **Tell us about other people’s reactions to girls being given pocket money. (**Find out about their family / friends)  **Is there any pressure on girls to share their money?** (find out the circumstances – who from, what is said, how do the girls react) |
| **Menstrual Issues**  We already know a lot about this so just concentrate on a) whether the schools are giving pads out – and if so how useful this is and whether they use their cash to buy sanitary pads / cup. | **Since being in the study has this made a difference to girls menstrual issues?** (Find out whether they are using their cash to buy pads or a cup – if so, how is this working out? We need to understand how this makes a difference to their lives. Do they have money left over?)  If they have bought a cup find out why and their experience of using it.  **Does your school give pads to girls?** (If so, find out the circumstances e.g how they do this, whether it is to all or some girls, on regular basis or only if they begin menstruating without having any protection etc and how helpful this is, what is good and what is not good about this) |
| **Relationships**  We want to find out whether being in the study and having pocket money affects girls relationships with boys and men. We are particularly interested in any changes in whether having pocket money means that girls don’t need to have transactional sex any more, or if it reduces the number of partners. | **Do you think being part of the study has made any difference to girls relationships with boys / men?** (find out what specific behaviours / what differences / what has caused them. Remember girls received puberty / hygiene training / soap / as well as a cup)  **Since being in the study do girls still need to have ‘boyfriends’ to buy other essential items …?** (find out what their reasons are and what sort of things they buy. Do they have one partner for this or many / has this changed since being in the study) |

**MIDLINE FGD SCHOOLGIRLS CASH + CUP GROUP**

| **Schooling**  Here we are trying to find out what girls hope to achieve by being at school and what the barriers to being educated are. We need to understand how we can help keep them in school. | **What have you liked about the study so far?**  **What have you not liked about the study?**  **Is there anything that has made it difficult for you, or girls like yourself, to remain at school?** (find out what this is, why it is a problem and what they think would help)  **What are the reasons that girls drop-out from school?** (If they discuss menstrual issues or money difficulties then probe the items in the sections on menstrual issues / Cash)  **What could help girls to stay in school?**  **Do you find it much different being in form 3 compared to last year in form 2?** (probe why so)  **Is there more pressure on you now that you are in form 3?** (probe to find what the pressures are – coursework / homework / family / friends / chores / relationships etc and how they affect the girls) |
| --- | --- |
| **School attendance and performance**  Here we want to find out if there have been any changes in drop out, absenteeism and performance since the girls were given cash or a cup. NB be aware that we know some of this already from previous FGDs so here the emphasis is on finding if there are any differences since the trial started and if so, why these have happened. | **Since the study started have there been any changes in girls dropping out from school?** (get them to describe any so that we understand why these have happened and whether / how this is because of getting pocket money or a cup)  **What about changes in girls being absent from school since starting in the study?** (get them to describe any so that we understand why these have happened – probe to find out if having pocket money or a cup has made a difference, how and why)  **Have girls ability to work in class, and to do well in lessons changed since the study started?** (Get them to describe any changes so that we understand why these have happened and whether it is due to having a pocket money or a cup)  **What makes it difficult for girls to do well in school**? (find out why and what can be done to improve things for girls) |
| **Cash**  So here we want to know if being given money help girls remaining at school or doing well at school, why and how this has happened. | **Has being given pocket money meant it is easier for girls to stay in school?** (If so, how and why )  **What about being absent from school – has this changed since being given pocket money** (If so, how and why)  **Has being given pocket money made any difference to girls lives generally?** (find out how and why / why not)  **Has it made any difference to the families of girls in the study** (find out how and why / why not)  **What sort of things do girls spend their pocket money on?**  **Do you think girls now have enough money for what they need?** (why / why not?) **What other things would they like to buy but don’t have enough money for**  **is it possible for girls to be able to save any pocket money?** (find out if this is easier than before they were in the study, and what they are saving for)  **Tell us about other people’s reactions to girls being given pocket money. (**Find out about their family / friends)  **Is there any pressure on girls to share their money?** (find out the circumstances – who from, what is said, how do the girls react)  **What support do you get from your family in coming to school?** (If they don’t then find out why) |
| **Cup**  We need to find out whether having cash and a cup is enough to keep girls at school and meet their needs, or whether just a cup or cash would be enough. | **Since being in the study, has this made a difference to girls menstrual issues?** (Find out whether they are using their cash to buy pads if so, how is this working out? We need to understand how this makes a difference to their lives. Do they have money left over?)  **What do girls think about the menstrual cup now?** (probe if girls use / why / not / any difficulties in using, if so, what are they? Is there anything that would help them to use/ Do they use for the whole time they are menstruating, if not / why not / when do they use or not.  **Do any girls prefer to use pads?** If so, **Are any girls using their pocket money to buy pads?** Find out how many girls (if any) do either. Find out why they prefer not to use the cup)  **Does your school give pads to girls?** (If so, find out the circumstances e.g how they do this, whether it is to all or some girls, on regular basis or only if they begin menstruating without having any protection etc and how helpful this is, what is good and what is not good about this)  **Do you know whether there are girls who have sold their cup?** (Was this their choice? Do many girls do this / why / who to? what was the money used for? what do they use for menstruation instead of a cup?).  **Do you know whether there are girls who have given away their cup?** (if so, probe why / was this their choice or were they put under pressure / was this given to family member or other? What do they use for menstruation instead of a cup?) |
| **Relationships**  We want to find out whether being in the study and having pocket money or a cup affects girls relationships with boys and men. We are particularly interested whether having a cup and / or pocket money makes any difference to girls need for transactional sex, or reduces the number of partners. | **Do you think being part of the study has made any difference to girls relationships with boys / men?** (find out what specific behaviours / what differences / what has caused them. Remember girls received puberty / hygiene training / soap / as well as a cup and cash)  **Since being in the study do girls still need to have ‘boyfriends’ to buy other essential items …?** (find out what their reasons are and what sort of things they buy. Do they have one partner or many – has this changed since being part of the study?) |
|  |  |
