# Supplementary material for: ‘You don’t have to sleep with a man to get how to survive’: Girl’s perceptions of an intervention study aimed at improving sexual and reproductive health and schooling outcomes
Source: PLOS Glob Public Health. 2022 Oct 13;2(10):e0000987. doi: 10.1371/journal.pgph.0000987 (PMC10021241; doi:10.1371/journal.pgph.0000987)
Supplement: Attachment — Submitted filename: Response to reviewers.docx [file pgph.0000987.s006.docx]

Journal Requirements:

Please include a complete copy of PLOS’ questionnaire on inclusivity in global research in your revised manuscript. Please upload a completed version of your questionnaire as Supporting Information when you resubmit your manuscript.

**This has been uploaded as Supporting Information as requested.**

Please amend your detailed Financial Disclosure statement. This is published with the article. It must therefore be completed in full sentences and contain the exact wording you wish to be published.

- State the initials, alongside each funding source, of each author to receive each grant.

**Please advise: The statement below is as we originally presented. We have stated the source of the grant and the grant holder. Her initials have been given. None of the other authors were grant holders for this study. We are unsure as to how you wish this to be amended. Thank you.**

***‘This work is part of the Cups or Cash for Girls randomised-controlled trial funded by the Joint Global Health Trials Initiative (UK-Medical Research Council/Department for International Development/Wellcome Trust/Department of Health and Social Care – https://www.ukri.org/opportunity/joint-global-health-trials/). Senior author PPH received the award, grant MR/N006046/1. The funders had no role in study design, data collection and analysis, decision to publish, or preparation of the manuscript’.***

Additional Editor Comments (if provided):

Based on the reviewers' comments, you will need to clarify the manner through which the interventions were administered and the FGD method used.
Regarding the sample selection, additional information on selection of girls from the sampled schools is required. Understanding the inclusion criteria, what approach was used to invite the girls to participate, and who and how specifically were they targeted?
A rationale for the use of FGDs as opposed to other qualitative methods is also required.
You will also need to respond to reviewers' critical concerns about respondent bias and correct minor grammatical errors.

**Thank you for these helpful suggestions. More detail has been incorporated, including response to concerns about respondent bias, and the grammatical errors amended. These are outlined in detail within the relevant reviewer section below.**

Reviewer 1

Abstract:
In the background section, could you include some information on factors known to be associated with your three main outcome variable (pregnancy, drop out, absenteeism)? Please include more information on the study population (e.g., age range, what country, what SES, other important descriptive variables) in the methods section. Tell us more about the FG methods (were the interviews semi-structured – when was baseline, midline, and study end completed)? What was the study duration?

**Unfortunately, while we agree that it would be informative to include such detail, we are constrained by the journal requirements that the abstract not exceed 300 words. We have considered what information we could delete in order to achieve this target but feel much of the current content is critical to describing the study. We have now included mention that the study is conducted among adolescent girls in a rural impoverished area of Kenya and that the discussion was semi-structured. However, to incorporate information on factors known to be associated with pregnancy, drop-out and absenteeism would require substantial increase in word count to do this with clarity. We did not include the specific times each round of FGD were held, nor study duration, as we noted that this information is not usually presented in other PLOS Global Health abstracts that we checked for consistency. However this is now included in the main body of the text.**

**Please note we are happy to amend if permission is given from the Editors for a lengthier abstract.**

General Language/Formatting:
I believe it is sub-Saharan (not Sub-Saharan), Also, your first LMIC abbreviation should include an ‘s’ as should STI’s and RTI’s. Also, “decision making at THE household and employment level”. Enrollment in line 98 is spelled wrong. Enroll in line 98 is spelled wrong. The comment that 4/10 girls complete secondary education needs a citation. Enrollment is spelled wrong throughout the manuscript and there are several typos. This sentence needs a citation: “Girls in rural Kenya report engaging in transactional sex to acquire necessities, including to fund their schooling needs.” Data is plural, so please change ‘data was’ to ‘data were’ and so forth throughout. I recommend using the terms boys and girls or men and women instead of sex-specific terms like male and female. The three supplementary files are not referenced in the text, but should be.

**We thank you for your attention to detail. We have amended the spellings as requested and changed to ‘American’ English spelling for enrolment. The reference for the World Bank indicators showing that 4 / 10 girls complete secondary education has been moved to the end of the sentence for clarity. The sentence regarding engaging in transactional sex has been amended using i.e to demonstrate that the citations 18-20 refer to both sentences. This now reads**

**‘*Girls in rural Kenya report engaging in transactional sex to acquire necessities, including to fund their schooling needs i.e. monies to pay for fees, purchase uniforms and acquire sanitary pads [******18-20******]***.

**We note that data is indeed plural and have amended as suggested. We have made some amendments regarding use of the terms male and female but feel there are some occasions where these sit better than using boys / men or girls and women e.g**

**‘*Noticeably, these relationships seemed to be alluded to in the context of male peers rather than emerging when asked about relationships with older men’.***

**We have therefore been mindful of this feedback and used with discretion. Thank you very much for picking up that we have not referenced the supplementary files in the text, these are now included in the method section where mention is made of the ‘Tools’.**

Introduction:
AGYW from SSA endure 2x the rate of morbidity and mortality compared to any other region? Do you mean here that AGYW in SSA have 2x the rate of M&M compared to AGYW outside SSA? Is this the overall M&M rate or related to SRH health risks?

**Yes, indeed we mean that AGYW have twice the rate of maternal mortality compared to AGYW outside of SSA. To provide clarity we have amended to**

***‘where they endure twice the mortality rate of AGYW in any other region’***

For ‘better employment chances” do you mean opportunities for employment?

**Thank you, this has been amended to**

**‘*greater potential for employment and higher wage earning capacity’***

There is complexity in poverty, retention in school, and pregnancy. Does poverty drive early marriage?

**We agree there is complexity in these relationships, however we would prefer to focus on the relationship between poverty and pregnancy, as these issues are key to our study, while noting that pregnant girls often drop out of school. There is minimal focus on marriage within our data, it was not a key driver to school drop-out nor pregnancy according to our participants, so that we feel further discussion would complicate the introduction, although we now mention this briefly in relation to the point below.**

Do schools allow girls to remain in school if pregnant?

**Thank you, this is a salient point. The following information with references, has been incorporated**

**‘*Recent legislative changes within many SSA countries now permit girls to remain in school if pregnant, yet social and cultural mores may discourage attendance among girls who are visibly pregnant, or from returning to school as a mother (11). A recent study in Kenya reported that pregnancy drove school drop-out which in turn led to early marriage (12)’***

Methods:
What does ‘issued termly’ mean?

**This has been amended to ‘*each school term’***

How was ‘80% school attendance’ measured?

**We have included the following description**

**‘*conditional upon 80% or greater attendance in the previous school term as measured using school registers alongside spot-checks by the research team. Financial literacy training was also provided’***

What was the general age range, as secondary school age can differ by educational system?

**We have included additional information on the main study trial and eligibility, including the participants age range.**

***‘The main study was a 4-arm cluster randomised controlled trial conducted in 96 secondary schools in rural western Kenya between 2017 and 2021, (described in detail elsewhere [******28******]). Schools were selected if they were day schools, included female students, and had approval from the principal. Schools were excluded if they only accepted full-board students, were special needs, or did not include female students. Schoolgirls were eligible if they were in the eligible study schools, in the selected school years (grades), had reached menarche, were not visibly pregnant, had no disability precluding participation, had parental consent and gave personal assent. At enrollment, the median participant age was 17.1 years (interquartile range [IQR] 16.3 to 18.0 years’.***

**Please note also the age range of our FGD participants is provided in Table 1.**

Were the same girls retained in each focus group for all three time points?

**No, we hope this is clarified by adding the following:**

**‘*231 girls participated in total: This comprised 79 at baseline, 77 midline and 75 at endline with no girl participating in more than one FGD***  ***in order not to overburden girls with too many study activities, and as recommended by our ethical review panels (See Table 1 for Group composition)’***

Results:
Please provide more interpretation or context before and after quotes. Why did the person say she couldn’t go back to school in the 3rd quote? Weren’t all girls in this study in school (due to the selection of randomizing schools)?

**We have included the following context to explain the above quote.**

**‘*Girls believed pregnancy and poverty in particular the lack of school fees - were the key drivers, with menstruation also frequently mentioned as a contributor. With one girl highlighting that if visible leakage of menstrual blood occurred to her it would seem so shameful she doubted she would be able to return to school***’.

**Please note that all girls participating in an FGD were in school at the time of their participation, however this did not preclude them from dropping out at any point after. In this instance the girl was only hypothesising how she might feel should that situation occur and that it would likely lead her to drop-out.**

Why were the mid and endline results presented in the same part of the results section? Was there no difference between immediately after and 2 years after the intervention?

**Yes, this is correct, while we found some small difference in the narratives over time, thematic analysis showed many similarities between mid and endline discussions. Furthermore, to include findings from baseline, midline and endline separately would have increased our already rather long results section to its’ detriment. We have now acknowledged this at the start of our results section as follows:**

‘***The results are presented initially by baseline findings to illustrate the views and experiences of girls prior to receipt of intervention, whilst findings from mid and endline are combined due to minimal differences in girls perceptions across these 2 time periods’.***

Discussion:
Selection bias likely existed since all participants were selected from schools, therefore they attended school. You are likely missing the most vulnerable because of this selection process. This study is also vulnerable to response bias, meaning the participants may have responded to what they thought the researchers wanted to hear.

**Yes we agree. Our research was a school-based intervention so we can only report on what girls attending school who participated thought and experienced – and verbalised to us, and may thus have been prone to desirability bias. We have tried to be very clear in our wording that any results were specific to these girls perceptions only, and indeed have not made any attempt to extrapolate such findings to a wider population. We have added a reminder to the readers of this point in the study limitations, along with expanding the point that our study was vulnerable to social desirability (or response) bias.**

**‘*The openness of discussion suggests that we were able to tap into the experiences of our participants. It may be, however, that this was also influenced by the gratitude girls felt in being part of a research study whereby they received at minimum, puberty and hygiene education. This may also have contributed to response bias whereby the participants say what they believe the researchers want them to say. However, much of the narrative pertaining to empowerment and thoughts around the interventions and their effects, emerged quite naturally through the discussions, as we concentrated initially on questioning around the material intervention and its influence on relationships and school outcomes. That some differences appeared to exist according to the different interventions provides a degree of confidence that social desirability responses may have existed but were not dominating discussions’.***

Reviewer 2

It appears the pre-intervention interviews explored the questions of interest among girls in general before any intervention/treatments were applied. If so this needs to be clearly spelt out.

**We have included the following information within the Methods – Current Qualitative study and hope that this clarifies this matter.**

***‘The FGDs were conducted at three different time points across the study. At baseline (pre-intervention) we explored girls’ perceptions of school, alongside risk factors and reasons for drop-out. We concentrated on issues found in the literature to influence schooling and drop-out, including relationships and menstruation. At midline (approximately one year after receiving the intervention) and endline (approximately 2 years after receiving intervention) we explored issues around girls’ perceptions of any impact the interventions had on their schooling and drop-out, again delving into potential influence on relationships and menstruation’.***

How exactly were the interventions/treatments administered? Were the interventions e.g. cash transfer administered to selected girls in particular schools or to all girls in selected schools? How were the girls who were selected for the FGDs subsequently selected? For example did girls or schools who received menstrual cups only constitute one homogenous group (Cup only) for the FGDs? it is important that these details are provided to guide the reader.

**We have now included the following paragraph within the section ‘Current Qualitative Study.**

**‘*Randomisation of the intervention took place at school level, so all eligible girls in the selected grades in that school received the same intervention provided that they met the criteria for study inclusion. Girls in the FGD were invited because of the intervention their school was allocated to. All participants in a cup group (with or without CT) were given a menstrual cup, but may not have used it. Similarly, girls in the CT group may not have received all of their monies as receipt was conditional upon 80% attendance in the***

The information provided in Table 1 seems to show that different schools and different girls were interviewed in the pre-intervention, mid-line and end-line focus group discussions. The table presents 24 different focus groups for the collective total of 24 FGDs that were conducted for this study. It is, however, not clear if the same group of girls were interviewed in the same groups at baseline, midline and end line or different groups of girls were interviewed at the different time points.

**Thank you, this point was also raised by reviewer 1 and we hope it has been clarified by the following addition**

***‘231 girls participated in total: This comprised 79 at baseline, 77 midline and 75 at endline with no girl participating in more than one FGD in order not to overburden girls with too many study activities, and as recommended by our ethical review panels (See Table 1 for Group composition)’.***

It will be helpful if the authors can provide a diagram or figure that shows; (1) how the girls were randomly assigned to the 4-arms, and (2) how the various treatments were assigned, and (3) how the FGDs were conducted according to treatment arms (for the 24 FGDs reported in Table 1).

**We have included a flow diagram to help guide the reader. Please see SI Flow Diagram 1.**

4. What informed the choice of menstrual cups rather than other menstrual products such as pads or tampons. While menstrual cups tend to be long lasting and reusable, usage is more difficult and less common. This could inform the findings of low use and non-use reported by the girls.

**We have included information on why the interventions were chosen (also CT) in the Methods section – Main Study Trial Design. This reads**

***‘The interventions were chosen on the basis of prior research which has demonstrated that provision of cash transfer can impact positively on school indicators and protect against some STI (10,30), whilst our previous feasibility study found the menstrual cup can reduce rates of reproductive tract and STI’s (31). Furthermore, girls provided with a cup (who had experienced using sanitary pads) appeared to express a preference for them over pads (32).***

Also, were the girls educated on how to use the menstrual cups as part of the study?

**Within the method section we have removed ‘*cup use training’* as the message seems to have been lost and replaced it with**

**‘all girls were provided with one Mooncup®, a reusable bell-shaped receptacle inserted to collect menstrual flow** ***together with training in safe cup use and care’.***

Given that menstrual cups seem to have made minimal impacts, would the authors recommend the provision of pads for example as this seems to be more common and user friendly for the girls?

**We do not agree that girls thought cups had a minimum effect. Girls reported that they made a difference to school absence. While a very salient point is made with regard to pads, we prefer not to cover in the discussion of this paper as we feel that it is slightly wider than the focus of the present paper. In addition, to do this justice we feel that it would require including a great deal more information. We prefer to concentrate our discussion on the points that are raised directly from this analysis and hope that this meets with your satisfaction.**

5. The authors mention using thematic analysis but the presentation of the results does not seem to follow particular themes. Rather the results are presented more along the lines of issues discussed in the various FGDs.

**We collected a great deal of data within the study and the analysis was indeed thematic, but we chose to explore and present here the key findings that would help us answer the research question addressed in this paper. We hope the following explanation which is now included in the methods section will help to clarify.**

***‘The FGDs covered topics wider than the research aim for the present paper, so from the framework we specifically chose the codes that would help us meet this aim. These covered schooling and outcomes, (drop-out, pregnancy, menstruation) as well as relationships and allowed us to compare and contrast across study arms as well as over the 3 timelines’.***

Minor formatting issues
1. Page 25, line 606 - Delete while
2. Page 27, line 647 - write IDetc as ID etc.

**These have been amended accordingly.**
